# Patient versus physician preferences for lipid‐lowering drug therapy: A discrete choice experiment

**DOI:** 10.1111/hex.14043

**Published:** 2024-04-08

**Authors:** Lingli Zhang, Jiali Chen, Zhaoliu Cao, Mengdie Zhang, Rui Ma, Pei Zhang, Guiqing Yao, Xin Li

**Affiliations:** ^1^ Department of Pharmaceutical Regulatory Science and Pharmacoeconomics, School of Pharmacy Nanjing Medical University Nanjing China; ^2^ Department of Health Policy, School of Health Policy and Management Nanjing Medical University Nanjing China; ^3^ Department of Pharmacy Nanjing City Qixia District Hospital Nanjing China; ^4^ Department of Cardiovascular Sciences and Leicester Clinical Trial Unit, College of Life Sciences University of Leicester Leicester UK; ^5^ Center for Global Health, School of Public Health Nanjing Medical University Nanjing China

**Keywords:** discrete choice experiment, lipid‐lowering drug, preferences

## Abstract

**Background:**

The emergence of proprotein convertase subtilisin/kexin type 9 inhibitors offered dyslipidemia patients an alternative to statins for lipid‐lowering treatment. Understanding patient and physician preferences for lipid‐lowering drugs may promote shared decision‐making and improve treatment outcomes.

**Methods:**

This study utilized an online discrete choice experiment (DCE) to assess the relative importance (RI) of six attributes related to lipid‐lowering drugs, including frequency of administration, mode of administration, reduction of low‐density lipoprotein cholesterol (LDL‐C) level, risk of myopathy, risk of liver damage, and out‐of‐pocket monthly cost. Respondents were recruited from dyslipidemia patients and cardiovascular physicians in China. A mixed logit model and latent class analysis were employed to estimate the preference coefficient, marginal willingness to pay (mWTP), and RI of attributes. Ethical approval has been obtained for this study.

**Results:**

A total of 708 patients and 507 physicians participated in the survey. Patients prioritized the ‘risk of liver damage’ (RI = 23.6%) with ‘mode of administration’ (RI = 19.2%) and ‘frequency of administration’ (RI = 18.8%) following closely. Contrarily, physicians prioritized the ‘reduction of LDL‐C level’ (RI = 33.5%), followed by ‘risk of liver damage’ (RI = 26.0%) and ‘risk of myopathy’ (RI = 16.1%). Patients placed a higher value on ‘frequency of administration’ (*p* < .001) and ‘mode of administration’ (*p* < .001) compared to physicians, while physicians valued ‘reduction of LDL‐C level’ (*p* < .001) and ‘risk of myopathy’ (*p* = .012) more than patients. Physicians exhibited higher mWTP than patients for all attributes except frequency and mode of administration. The LCA revealed three distinct patient classes: focus on oral administration, focus on hepatic safety and frequency and focus on hepatic safety and cost. Likewise, three physician classes were identified: frequency‐insensitive, efficacy‐focused and safety‐focused.

**Conclusions:**

The preferences for lipid‐lowering drug therapy differed between patients and physicians in China. Physicians should take into account patients' preferences and provide personalized treatment when they formulate lipid‐lowering treatment plans.

**Patient or Public Contribution:**

Patients participated in the questionnaire design process. They engaged in a focus group discussion to determine attributes and levels and also participated in a pilot survey to assess the comprehensibility of the questionnaires. Additionally, patients were involved in the DCE survey to express their preferences. The findings of patient preference for lipid‐lowering drug therapy will promote shared decision‐making and optimize the treatment regimen.

## 1 INTRODUCTION

Elevated blood lipid levels, particularly low‐density lipoprotein cholesterol (LDL‐C), are major risk factors for atherosclerotic cardiovascular disease (ASCVD), including myocardial infarction and stroke.^1^ Despite the well‐established benefits of lipid‐lowering drug therapy in preventing cardiovascular events, nonadherence to treatment recommendations is common among patients worldwide.^2^ In China, the prevalence of dyslipidemia is rapidly increasing, with an overall prevalence of more than 40%.^3^ However, only 14.1% of established ASCVD patients requiring lipid‐lowering drugs received adequate treatment.^4^ Thus, ensuring adequate treatment and control of dyslipidemia has become a priority in public health.

Several lipid‐lowering drugs are available for patients with dyslipidemia. Statins are the primary therapy for reducing cholesterol levels. However, not all patients with statins can achieve their LDL‐C targets even with higher dosages, and side effects including liver damage and myopathy may occur. Proprotein convertase subtilisin/kexin type 9 (PCSK9) inhibitors are emergent lipid‐lowering drugs that have been proven to be a safe and effective treatment alternative.^5^ However, the nonoral route of administration and high cost limited their widespread use. The Chinese government has incorporated two PCSK9 inhibitors into the drug reimbursement list under the national drug price negotiation policy, aiming to alleviate the financial burden on patients.^6^

The guidelines for blood cholesterol management in the US advocate for shared decision‐making between physicians and patients to improve treatment adherence.^7^ However, shared decision‐making is uncommon in China, and patient preferences are often ignored.^8^ Notably, numerous studies have revealed different preferences between patients and healthcare professionals in treatment options for various medical conditions.^9^, ^10^, ^11^, ^12^ For example, oncologists may prioritize overall survival time, while cancer patients may value physical functioning status and pain management more.^9^ Similarly, neurologists prioritize seizure control over patients undergoing antiepileptic drug therapy.^10^ These differences in preferences have also been observed in other areas, such as treatments for bone metastases and preventive treatments for migraines.^11^, ^12^ However, to our knowledge, no published literature has explored the differences in patient and physician preferences for lipid‐lowing drugs. Consequently, it is crucial to evaluate and compare patient preferences for lipid‐lowering drugs with those of physicians.

Discrete choice experiment (DCE) is a quantitative approach which is used to elicit individual preferences through trade‐offs among alternatives.^13^ It has gained popularity for quantifying preferences in drug therapy, including cancer treatment,^9^, ^14^ antiepileptic drugs,^10^ antihyperglycemic medications,^15^ asthma medications,^16^ treatments for rheumatoid arthritis^17^ and anticoagulant therapy.^18^ These DCE studies have provided insights into the preferences of respondents for treatment options. For instance, cancer patients may assign greater importance to outcome measures (such as overall survival) and cost attributes compared to process attributes.^14^ These preference evidence can inform clinical decision‐making and facilitate shared decision‐making, ultimately leading to improved healthcare outcomes. However, the literature review has identified only one study that explored patient preferences for lipid‐lowering drugs using DCE,^19^ and it did not include out‐of‐pocket cost as an attribute since the German healthcare system does not rely on co‐payments. Conversely, out‐of‐pocket cost was a key factor influencing treatment decisions in China.^15^ In addition, the study did not explore physician preferences.

Therefore, the present study aimed to quantify and compare the preferences of patients and physicians regarding lipid‐lowering drug therapy. The study sought to answer the following research questions: (1) What attributes do patients and physicians consider essential for lipid‐lowering drug therapy? (2) Are there differences in lipid‐lowering drug therapy preferences between patients and physicians? (3) Do patient and physician subgroups exhibit distinct preference patterns? The findings from this study will help physicians tailor lipid‐lowering drug regimens to better meet patient needs and enhance drug adherence.

## 2 METHODS

The development of DCE in this study adhered to the recommended research practices of the International Society for Pharmacoeconomics and Outcomes Research.^20^ Key steps included defining the research question, identifying attributes and levels, constructing choice sets, collecting data and analyzing data.

### 2.1 Identifying attributes and levels

The attributes of DCE in the study were based on a comprehensive literature review and focus group discussion. Our literature review on DCEs for drug treatment preferences revealed that these attributes commonly encompass outcome (e.g., efficacy and adverse effects), process (e.g., mode of administration) and cost.^9^, ^10^, ^14^, ^15^, ^16^, ^17^, ^18^ Additionally, we conducted a literature review to gain insights into the characteristics of lipid‐lowering drugs, and the search strategy can be found in Supporting Information S1: Appendix 1. Thus, we identified 10 candidate attributes based on the literature review (Supporting Information S1: Appendix 2). Then, a focus group comprising 10 patients and 10 physicians, all of whom possessed knowledge and experience with lipid‐lowering drugs, was assembled through convenience sampling at a local hospital. Based on the feedback from the focus group discussion, four attributes were identified as less significant and subsequently excluded (Supporting Information S1: Appendix 2). Ultimately, six attributes were incorporated into the DCE: frequency of administration, mode of administration, reduction of LDL‐C level, risk of myopathy, risk of liver damage and out‐of‐pocket monthly cost.

Establishing levels of each attribute was based on literature review and expert opinion. More detailed information about levels and corresponding references can be found in Supporting Information S1: Appendix 3. The mode of administration had two levels: oral and subcutaneous injection. The remaining attributes used the highest and lowest values reported in the literature as the upper and baseline levels, respectively, and their ranges were averaged to create three or four levels. Patient preference experts were also consulted to validate the rationality of the attributes and levels we established. Table 1 demonstrates the six attributes with corresponding levels.

**Table 1:** Attributes and levels included in the discrete choice experiment.

Attributes	Levels
Frequency of administration	Once a day
	Once a week
	Once every 2 weeks
	Once a month
Mode of administration	Oral
	Subcutaneous injection
Reduction of LDL‐C level	High (by 60%)
	Medium (by 35%)
	Low (by 10%)
Risk of myopathy	0%
	2.5%
	5%
Risk of liver damage	0%
	2.5%
	5%
Out‐of‐pocket monthly cost (CNY)	0
	100
	200

Abbreviations: CNY, Chinese yuan; LDL‐C, low‐density lipoprotein cholesterol.

### 2.2 Experiment design

**Figure 1:**
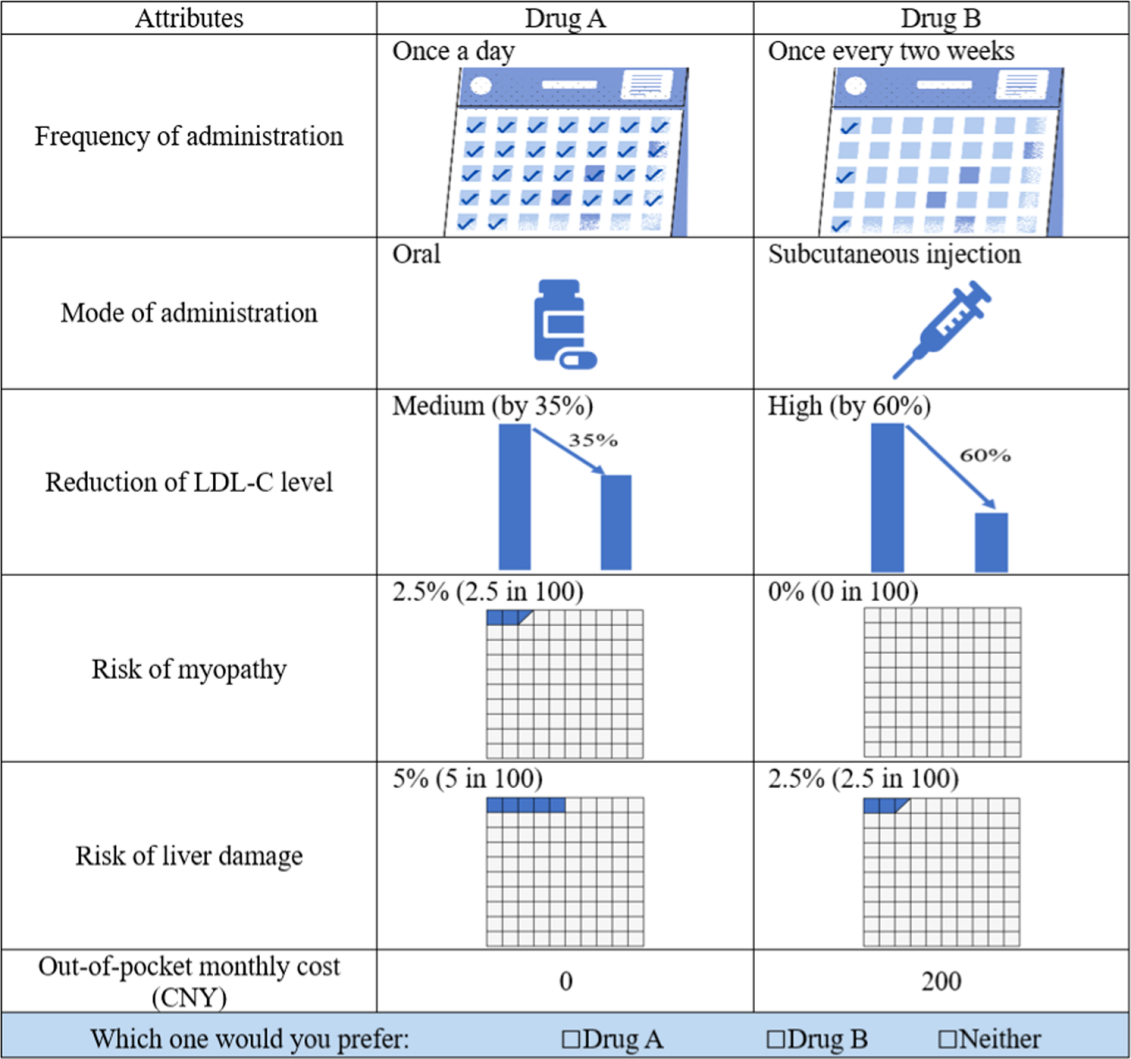
An example of a choice set. CNY, Chinese yuan; LDL‐C, low‐density lipoprotein cholesterol.

Bayesian D‐optimal design, a fractional factorial design, was employed to generate choice sets using SAS JMP (version Pro 14).^21^ The prior means and variances required in the design were determined through a face‐to‐face pilot survey involving 30 dyslipidemia patients and 30 cardiovascular physicians and was shown in Supporting Information S1: Appendix 4. For each group, 16 choice sets were generated and divided into two blocks. We then randomly assigned an equal number of respondents to each block. Each choice set comprised two scenarios with different attribute levels and an ‘opt‐out’ option. The ‘opt‐out’ option could reduce the bias from a forced‐choice design by allowing participants to choose neither drug. Choice sets were presented without labels to ensure attribute attention. Attribute order within choice sets was randomized to mitigate order effects. Graphical representations in attribute‐level descriptions aided decision‐making. An example of a choice set is shown in Figure 1.

Both the patient and physician questionnaires comprised three sections. The first section collected sociodemographic data and relevant patient health status or physician practice information. The second section introduced the attributes and levels and presented respondents with a comprehension‐checking question comprising dominant options, and only those who responded accurately proceeded to the next section. The third section included nine choice sets, eight used for data analysis and one as a duplicate to assess response consistency for internal validation.

### 2.3 Survey

We conducted a cross‐sectional online survey to administer the questionnaire. The sample size was determined using Orme's formula (*N* > 500 × *c*/(*t *× *a*)), which required a minimum of 125 respondents (with a maximum of four levels, eight choice sets and two alternatives). To ensure robust statistical estimates and enable heterogeneity analysis, we increased the sample size by a factor of four. Therefore, our target sample size was set at 500. We collaborated with national dyslipidemia patient organizations and cardiovascular physician associations to recruit respondents. These members were from diverse cities nationwide and exhibited variations in relevant factors, thereby potentially providing an adequate and representative sample for the online survey. Invitations were sent randomly to their members via email or the WeChat platform until the target sample size was reached. Recruitment quotas, including gender and disease status, were imposed to achieve a balanced composition of respondents. The inclusion criteria were dyslipidemia patients or physicians practicing cardiovascular medicine who had used or prescribed lipid‐lowering drugs within the past 6 months, were at least 18 years old and had no communication barriers. Potential respondents completed a screening questionnaire to determine study eligibility. Respondents who completed the survey received a ‘red packet’ (equivalent to $3) on WeChat as a monetary incentive. The online questionnaire was designed and generated on the Questionnaire Star website. All respondents were instructed to answer all questions in the questionnaire, ensuring no missing data. Respondents who completed the survey in less than 3 min (the minimum reasonable time based on the pilot survey) and those who made inconsistent selections in repeated choice sets were excluded from the data analysis. Data collection took place anonymously from July to November 2022.

### 2.4 Statistical analysis

Descriptive statistics were used to analyze respondent characteristics. A mixed logit model and latent class analysis were employed to estimate preferences. The ‘out‐of‐pocket monthly cost’ attribute was continuously coded to calculate marginal willingness to pay (mWTP), while dummy coding was applied to the remaining attributes. The alternative specific constants were also considered in these models. The random parameters were estimated using 500 standard Halton sequences. mWTP was calculated by dividing the coefficients of the other attribute levels by the ‘out‐of‐pocket monthly cost’ coefficients. The mWTP was reported in US dollars, using the 2022 exchange rate of 0.14879 US dollars to Chinese yuan (CNY) by the Central Bank of China. The relative importance (RI) of each attribute was determined by dividing the difference between the coefficients of the best and worst levels by the sum of all attribute differences. Independent *t* tests were used to assess whether the RI of attributes differed between patients and physicians. We constructed LCA models with 1 to 4 latent classes and assessed model fit indices (including Akaike information criterion [AIC], Bayesian information criteria [BIC] and log‐likelihood), model simplicity and clinical interpretability to identify the most appropriate number of latent classes. Lower values of AIC, BIC and log‐likelihood indicate better model fit. The chi‐square test examined variations in individual characteristics across different classes. In sensitivity analyses, the cost variable was treated as categorical using dummy codes instead of continuous variables with linearity, to accurately capture the impact of all three levels on respondent preference. Statistical significance was set at *p* < .05 (two‐sided), and 95% confidence intervals (CIs) were calculated using the bootstrap method. All statistical analyses were performed using Stata software (version 16).

## 3 RESULTS

### 3.1 Characteristics of respondent

The survey included 708 patients and 507 physicians. Table 2 presents the key characteristics of the patients and physicians. Among the patients, 48.16% were male, 35.45% were aged between 40 and 50 years, 76.55% had received a college education, 42.51% reported an annual income ranging from 80,000 to 150,000 CNY and 77.54% had Urban Employees Basic Medical Insurance. Regarding their disease, 35.73% reported the latest LDL‐C level ranging from 2.6 to 3.4 mmol/L, 27.26% had a duration of hyperlipidemia between 1 and 2 years, 54.66% were currently undergoing statin monotherapy, 84.18% reported comorbidities such as hypertension and diabetes and 50.85% reported fair health status. Among the physicians, 47.53% were male, 58.58% were aged between 30 and 40 years, 72.39% held a master's degree or above and 42.41% were attending physicians. Of these, 11 patients and 12 physicians were excluded for completing the survey before 3 min, and 70 patients and 75 physicians were excluded due to inconsistent selections in repeated choice sets, leaving 627 patients and 420 physicians in the final sample.

**Table 2:** Characteristics of respondents.

Characteristic	No. (%) of patients (*n* = 708)	No. (%) of physicians (*n* = 507)
Gender		
Male	341 (48.16)	241 (47.53)
Female	367 (51.84)	266 (52.47)
Age (years)		
18–30	111 (15.68)	174 (34.32)
30–40	194 (27.40)	297 (58.58)
40–50	251 (35.45)	34 (6.71)
50–60	135 (19.07)	2 (0.39)
>60	17 (2.30)	0 (0)
Education		
Junior high school or below	13 (1.84)	0 (0)
Senior high school	111 (15.68)	0 (0)
College	542 (76.55)	140 (27.61)
Master or above	42 (5.93)	367 (72.39)
Region		
Eastern	445 (62.85)	297 (58.58)
Central	163 (23.02)	116 (22.88)
Western	100 (14.12)	94 (18.54)
Individual income (CNY/year)		NA
≤30,000	26 (3.67)	
30,000–80,000	155 (21.89)	
80,000–150,000	301 (42.51)	
150,000–300,000	184 (25.99)	
>300,000	42 (5.93)	
Insurance		NA
Urban employees basic medical insurance	549 (77.54)	
Urban and rural residents basic medical insurance	140 (19.77)	
Free medical care	14 (1.98)	
Commercial insurance	4 (0.56)	
None	1 (0.14)	
Latest LDL‐C level (mmol/L)		NA
≤1.8	16 (2.26)	
1.8–2.6	200 (28.25)	
2.6–3.4	253 (35.73)	
3.4–4.9	131 (18.5)	
>4.9	20 (2.82)	
Not sure	88 (12.43)	
Duration of hyperlipidemia (years)		NA
≤0.5	126 (17.8)	
0.5–1	176 (24.86)	
1–2	193 (27.26)	
2–5	158 (22.32)	
5–10	44 (6.21)	
10–15	9 (1.27)	
>15	2 (0.28)	
Current treatment		NA
Statin monotherapy	387 (54.66)	
Nonstatin monotherapy	152 (21.47)	
Combination therapy containing a statin	154 (21.75)	
Other	15 (2.12)	
Comorbidities		NA
Yes	596 (84.18)	
No	112 (15.82)	
Patient‐reported health status		NA
Very good	11 (1.55)	
Good	128 (18.08)	
Fair	360 (50.85)	
Poor	193 (27.26)	
Very poor	16 (2.26)	
Academic title	NA	
Resident physician		181 (35.7)
Attending physician		215 (42.41)
Associate chief physician		76 (14.99)
Chief physician		35 (6.9)
Hospital level	NA	
Grade‐A tertiary		261 (51.48)
Grade‐B tertiary		92 (18.15)
Grade‐C tertiary		67 (13.21)
Secondary		75 (14.79)
Primary		12 (2.37)

Abbreviations: CNY, Chinese Yuan; LDL‐C, low‐density lipoprotein cholesterol; NA, not applicable.

### 3.2 Preference estimate by mixed logit model

Significant coefficients were observed for all attributes and levels within the patient population (*p* < .001), indicating that all levels influenced patient preferences significantly (Table 3). The decision‐making of physicians was significantly impacted by all attribute levels (*p* < .05), except for the frequency of administration between ‘once a week’ and ‘once a day’ (*p* = .80). The observed positive and negative values of the estimated coefficient aligned with our expectations, showing negative preferences among patients and physicians for subcutaneous injection, the risk of myopathy and liver damage, and higher costs while expressing a positive preference for less frequent administration and lower LDL‐C levels. Better levels were generally more preferred than objectively worse levels, except for the patient preference for ‘once every two weeks’ administration frequency, which was slightly less preferred than ‘once a week’ (0.76 vs. 0.88). In addition, both patients and physicians exhibited preference heterogeneity across several levels (*p* < .001 for SD).

**Table 3:** Patients' and Physicians' preferences and mWTP (US$/month) estimated by a mixed logit model.

Attribute and level	Patient					Physician				
	Coefficient (95% CI)	*p* Value	SD (95% CI)	SD *p* Value	mWTP (95% CI, US$/month)	Coefficient (95% CI)	*p* Value	SD (95% CI)	SD *p* Value	mWTP (95% CI, US$/month)
*Frequency of administration (ref: once a day)*										
Once a week	0.88 (0.69, 1.07)	<.001***	0.07 (−0.41, 0.55)	.78	26.82 (19.68, 33.96)	0.02 (−0.14, 0.18)	.80	0.06 (−0.37, 0.49)	.78	1.74 (−11.45, 14.93)
Once every 2 weeks	0.76 (0.57, 0.96)	<.001***	−1.07 (−1.36, −0.78)	<.001***	23.31 (16.25, 30.38)	0.19 (0.01, 0.36)	.04*	0.03 (−0.91, 0.97)	.95	15.02 (1.10, 28.94)
Once a month	1.30 (1.07, 1.53)	<.001***	0.99 (0.70, 1.28)	<.001***	39.64 (30.43, 48.85)	0.20 (0.02, 0.39)	.03*	0.66 (0.37, 0.94)	<.001***	16.50 (1.08, 31.91)
*Mode of administration (ref: oral)*										
Subcutaneous injection	−1.33 (−1.57, −1.10)	<.001***	1.76 (1.55, 1.97)	<.001***	−40.67 (−49.68, −31.67)	−0.26 (−0.40, −0.13)	<.001***	0.73 (0.56, 0.90)	<.001***	−21.19 (−34.36, −8.02)
*Reduction of LDL‐C level (ref: 10%)*										
35%	0.82 (0.66, 0.99)	<.001***	−0.78 (−1.05, −0.51)	<.001***	25.09 (18.49, 31.70)	0.39 (0.18, 0.61)	<.001***	−0.36 (−0.83, 0.12)	.14	31.89 (13.10, 50.68)
60%	1.00 (0.75, 1.24)	<.001***	1.96 (1.70, 2.23)	<.001***	30.40 (21.78, 39.01)	1.14 (0.90, 1.37)	<.001***	1.54 (1.31, 1.78)	<.001***	92.31 (51.08, 133.54)
*Risk of myopathy (ref: 0%)*										
2.5%	−0.42 (−0.57, −0.27)	<.001***	0.25 (−0.05, 0.56)	.11	−12.75 (−18.10, −7.41)	−0.33 (−0.52, −0.14)	<.001***	0.70 (0.47, 0.92)	<.001***	−26.94 (−43.10, −10.78)
5%	−0.69 (−0.85, −0.52)	<.001***	0.87 (0.62, 1.12)	<.001***	−20.97 (−27.21, −14.73)	−0.55 (−0.73, −0.36)	<.001***	0.81 (0.60, 1.02)	<.001***	−44.31 (−66.98, −21.64)
*Risk of liver damage (ref: 0%)*										
2.5%	−1.11 (−1.30, −0.92)	<.001***	1.51 (1.30, 1.71)	<.001***	−33.90 (−42.29, −25.50)	−0.64 (−0.79, −0.48)	<.001***	0.24 (−0.13, 0.62)	.20	−51.54 (−77.40, −25.68)
5%	−1.63 (−1.87, −1.40)	<.001***	1.90 (1.63, 2.17)	<.001***	−49.89 (−60.77, −39.00)	−0.89 (−1.07, −0.70)	<.001***	0.75 (0.52, 0.98)	<.001***	−71.88 (−103.90, −39.87)
Out‐of‐pocket monthly cost	−0.0049 (−0.0058, −0.0039)	<.001***	NA	NA	NA	−0.0018 (−0.0027, −0.0010)	<.001***	NA	NA	NA
*Model specification*										
Log‐likelihood	−4070.98					−2856.38				
AIC	8185.95					5756.76				
BIC	8353.57					5915.56				

Abbreviations: AIC, Akaike information criterion; BIC, Bayesian information criterion; CI, confidence interval; LDL‐C, low‐density lipoprotein cholesterol; mWTP, marginal willingness to pay; NA, not applicable; ref, reference.

*** *p* < .001

* *p* < .05.

Higher mWTP values reflect a greater willingness to pay for desirable attributes or levels. Physicians showed higher mWTP values than patients for all attributes except for frequency and mode of administration (Table 3). Patients had an mWTP of US$30.40 per month for ‘reduction of LDL‐C levels’ ranging from 10% to 60%, while physicians had an mWTP of US$92.31 per month. Conversely, patients had an mWTP of US$39.64 per month for ‘frequency of administration’ ranging from once a day to once a month, while physicians had an mWTP of only US$16.50 per month.

**Figure 2:**
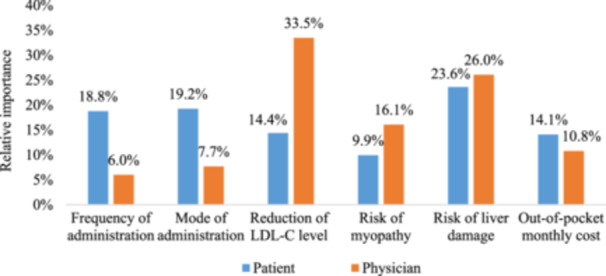
Relative importance of attributes to patients and physicians.

Figure 2 presents the RI of each attribute for patients and physicians. Patients prioritized the ‘risk of liver damage’ as the most significant attribute (RI = 23.6%). ‘Mode of administration’ (RI = 19.2%) and ‘frequency of administration’ (RI = 18.8%) were the subsequent dominant factors for patients. The importance of ‘reduction of LDL‐C level’ and ‘out‐of‐pocket monthly cost’ was similar for patients (RI = 14.4% and 14.1%). Among the six attributes, the ‘risk of myopathy’ (RI = 9.9%) had the lowest importance for patients. Conversely, physicians ranked ‘reduction of LDL‐C level’ (RI = 33.5%) as their highest priority, followed by ‘risk of liver damage’ (RI = 26.0%), ‘risk of myopathy’ (RI = 16.1%) and ‘out‐of‐pocket monthly cost’ (RI = 10.8%). The attributes of ‘mode of administration’ and ‘frequency of administration’, which ranked second and third in priority for patients, held lesser importance for physicians (RI = 7.7% and 6.0%). Significant differences were observed in the RI values of four attributes between patients and physicians. Patients placed higher value on ‘frequency of administration’ (*p* < .001) and ‘mode of administration’ (*p* < .001) compared to physicians, whereas physicians valued ‘reduction of LDL‐C level’ (*p* < .001) and ‘risk of myopathy’ (*p* = .012) more than patients. There were no significant differences in the RI values of ‘risk of liver damage’ (*p* = .37) and ‘out‐of‐pocket monthly cost’ (*p* = .17) between patients and physicians.

### 3.3 Preference heterogeneity

**Figure 3:**
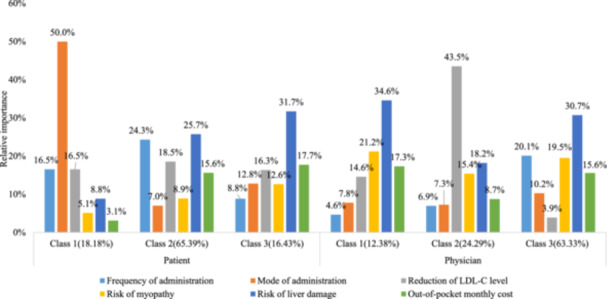
Relative importance of attributes to patients and physicians, separated by class. LDL‐C, low‐density lipoprotein cholesterol.

As the number of latent classes increased from one to four, the model fit indices consistently decreased. However, it became difficult to explain the characteristics of each class with four classes. Therefore, we concluded that a three‐class model was the most appropriate for both patients and physicians. The patient preferences, divided into three classes, are presented in Figure 3 and Supporting Information S1: Appendix 5. Class 1 patients (18.18%) prioritized oral administration (RI = 50%) and showed indifference towards the out‐of‐pocket monthly cost (*p* = .48). Class 2 patients (65.39%) were more inclined towards a lower frequency of administration than other classes, although the RI (24.3%) remained lower than that for the ‘risk of liver damage’ (RI = 25.7%). Class 3 patients (16.43%) demonstrated the highest preference for avoiding the ‘risk of liver damage’ (RI = 31.7%). However, they demonstrated a significantly diminished preference for frequency (RI = 8.8%) compared to class 2 patients. Additionally, the second highest ranking was given to ‘out‐of‐pocket monthly cost’ (RI = 17.3%). Among the three patient classes (Table 4), significant differences were observed in the age distribution (*p* = .02). Class 1 and class 2 had a higher proportion (31.58% and 29.27%) of patients aged 30–40 years and lower proportions (30.70% and 33.17%) of patients aged 40–50 years compared to class 3 (15.53% and 49.51%, respectively). Another notable difference was in patient‐reported health status (*p* = .004). Class 1 had a higher proportion of patients (66.67%) rating their health as ‘fair’ compared to class 2 (46.10%), while a lower percentage of class 1 patients (14.91%) rated their health as ‘poor’ compared to class 2 patients (30.73%).

**Table 4:** Characteristics of three patient classes.

Characteristic	Class 1 (*n* = 114)	Class 2 (*n* = 410)	Class 3 (*n* = 103)	*p* Value
	No. (%) of patients	No. (%) of patients	No. (%) of patients	
Gender				.57
Male	60 (52.63)	194 (47.32)	48 (46.60)	
Female	54 (47.37)	216 (52.68)	55 (53.40)	
Age (years)				.02*
18–30	14 (12.28)	72 (17.56)	13 (12.62)	
30–40	36 (31.58)	120 (29.27)	16 (15.53)	
40–50	35 (30.70)	136 (33.17)	51 (49.51)	
50–60	27 (23.68)	74 (18.05)	18 (17.48)	
>60	2 (1.75)	8 (1.95)	5 (4.85)	
Education				.07
Junior high school or below	5 (4.39)	6 (1.46)	1 (0.97)	
Senior high school	11 (9.65)	64 (15.61)	23 (22.33)	
College	89 (78.07)	316 (77.07)	75 (72.82)	
Postgraduate or above	9 (7.89)	24 (5.85)	4 (3.88)	
Individual income (CNY/year)				.53
≤30,000	3 (2.63)	16 (3.90)	4 (3.88)	
30,000–80,000	20 (17.54)	95 (23.17)	22 (21.36)	
80,000–150,000	47 (41.23)	171 (41.71)	49 (47.57)	
150,000–300,000	39 (34.21)	102 (24.88)	22 (21.36)	
>300,000	5 (4.39)	26 (6.34)	6 (5.83)	
Insurance				.19
Urban employee basic medical insurance	82 (71.93)	321 (78.29)	83 (80.58)	
Urban and rural resident basic medical insurance	27 (23.68)	79 (19.27)	18 (17.48)	
Free medical care	2 (1.75)	8 (1.95)	2 (1.94)	
Commercial insurance	3 (2.63)	1 (0.24)	0 (0)	
None	0 (0)	1 (0.24)	0 (0)	
Latest LDL‐C level (mmol/L)				.53
≤1.8	3 (2.63)	10 (2.44)	1 (0.97)	
1.8–2.6	32 (28.07)	120 (29.27)	25 (24.27)	
2.6–3.4	41 (35.96)	145 (35.37)	38 (36.89)	
3.4–4.9	14 (12.28)	81 (19.76)	21 (20.39)	
>4.9	5 (4.39)	9 (2.20)	4 (3.88)	
Not sure	19 (16.67)	45 (10.98)	14 (13.59)	
Duration of hyperlipidemia (years)				.22
≤0.5	4 (3.51)	37 (9.02)	10 (9.71)	
0.5–1	25 (21.93)	76 (18.54)	17 (16.50)	
1–2	32 (28.07)	111 (27.07)	29 (28.16)	
2–5	35 (30.70)	138 (33.66)	32 (31.07)	
5–10	13 (11.40)	41 (10.00)	9 (8.74)	
10–15	5 (4.39)	6 (1.46)	4 (3.88)	
>15	0 (0)	1 (0.24)	2 (1.94)	
Current treatment				.75
Statin monotherapy	67 (58.77)	219 (53.41)	57 (55.34)	
Nonstatin monotherapy	23 (20.18)	91 (22.20)	21 (20.39)	
Combination therapy containing statin	23 (20.18)	92 (22.44)	21 (20.39)	
Other	1 (0.88)	8 (1.95)	4 (3.88)	
Comorbidities				.22
Yes	96 (84.21)	351 (85.61)	81 (78.64)	
No	18 (15.79)	59 (14.39)	22 (21.36)	
Patient‐reported health status				.004**
Very good	0 (0)	10 (2.44)	0 (0)	
Good	20 (17.54)	74 (18.05)	19 (18.45)	
Fair	76 (66.67)	189 (46.10)	54 (52.43)	
Poor	17 (14.91)	126 (30.73)	28 (27.18)	
Very poor	1 (0.88)	11 (2.68)	2 (1.94)	
Region				.77
East	71 (62.28)	262 (63.90)	61 (59.22)	
Central	29 (25.44)	91 (22.20)	24 (23.30)	
West	14 (12.28)	57 (13.90)	18 (17.48)	

Abbreviations: CNY, Chinese Yuan; LDL‐C, low‐density lipoprotein cholesterol.

* *p *< .05

** *p *< .01.

Based on the findings presented in Figure 3 and Supporting Information S1: Appendix 6, it can be observed that class 1 physicians (12.38%) assigned the highest importance to the ‘risk of liver damage’ with a RI of 34.6% while considering ‘frequency of administration’ as least significant (*p* > .05). Class 2 physicians (24.29%) significantly emphasized ‘reduction of LDL‐C level’ with a RI of 43.5%, surpassing the other two classes. Class 3 physicians (63.33%) ranked ‘risk of liver damage’ as the top priority with an RI of 30.7%, consistent with class 1 physicians, but differed in ranking ‘frequency of administration’ second (RI = 20.1%). Supporting Information S1: Appendix 7 showed the physician characteristics across the three classes, and the differences across the classes were statistically insignificant. Additionally, the AIC and BIC values of the LCA model (Supporting Information S1: Appendices 5 and 6) were smaller than those of the mixed logit model (Table 3) for both patients and physicians, implying that the LCA model was the better fitting model.

### 3.4 Sensitivity analysis

Treating cost as a categorical variable yielded results (Supporting Information S1: Appendices 8 and 9) consistent with the main analysis. The primary concern of patients remained the ‘risk of liver damage’, and physicians prioritized the ‘reduction of LDL‐C levels’. A minor variation from the main analysis was the ranking of cost by patients, which surpassed the ‘reduction of LDL‐C level’, although the RI remained comparable (15.0% vs. 14.1%).

## 4 DISCUSSIONS

The present study provided empirical evidence of differences in patient and physician preferences for lipid‐lowering drug therapy for the first time. We found that patients prioritized avoiding the risk of liver damage, whereas physicians prioritized lowering LDL‐C levels. Patient preferences for oral administration and low frequency of administration were underestimated by physicians. Furthermore, the study identified three distinct preference classes among both patients and physicians.

We implemented several measures to ensure the quality of the questionnaire data. First, we conducted a pilot study to ensure the comprehensibility of the questionnaire. Second, we conducted a comprehension test before the choice tasks to ensure respondents' understanding. Third, we performed an internal validity test to exclude questionnaires with inconsistent answers in duplicate choice sets. Lastly, we excluded questionnaires that were completed too quickly.

Mixed logit and conditional logit models are frequently employed in econometric analyses of DCEs in the field of health economics.^22^ In this study, the mixed logit model was chosen because it can effectively account for unobserved preference heterogeneity among respondents compared to the classical conditional logit model.^23^ The mixed logit model, also known as a random‐parameters logit model, explicitly assumes the existence of a preference weight distribution across the sample, reflecting variations in preferences among respondents. Unlike the conditional logit model, which only estimates a series of coefficients representing the average preference weights of the attribute levels, the mixed logit model provides estimates for both the average effect and a standard deviation effect. Additionally, this study used the bootstrap method to calculate CIs for coefficients in the mixed logit model and LCA. Previous research has demonstrated the appropriateness of the bootstrap method in DCE when unobserved heterogeneity is suspected in the data, as it does not depend on any assumptions about the distribution of the coefficients.^24^

Compared with the dyslipidemia prevalence study conducted in China,^4^ our study observed similar gender distribution, health insurance status, medical history and treatment regimen among the dyslipidemia patients. However, our patients were relatively younger with higher education levels and income. These differences may be attributed to the challenges faced in conducting online surveys and DCE among older adults. Despite our efforts to recruit older patients, the advanced age of certain participants was often accompanied by reduced cognitive ability, making it difficult to complete online DCE. Similar online DCE studies have also reported relatively younger mean ages in their respondent.^25^, ^26^ Moreover, the alignment of the prioritization of the six attributes among patients aged 50 years and above was congruent with that of the entire patient sample (Supporting Information S1: Appendix 10), thereby affirming the reliability of our findings.

Our study revealed differences in preferences between patients and physicians for lipid‐lowering drug therapy, consistent with previous findings in cancer and neurological disease.^9^, ^10^, ^11^, ^12^ Physicians prioritized the ‘reduction of LDL‐C level’ and paid insufficient attention to patient preferences for oral administration and infrequent dosing. These findings emphasize the importance of recognizing divergent preferences between patients and physicians, promoting shared decision‐making, and involving patients in developing treatment plans.

A previous study conducted in Germany on therapy preferences for lipid‐lowering drugs highlighted the significance of the attributes ‘reduction of LDL‐C level’, ‘risk of myopathy’ and ‘frequency of apheresis’ among patients with severe hypercholesterolaemia.^19^ However, since lipoprotein apheresis is infrequently used in China, our study did not include ‘frequency of apheresis’ as an attribute. Considering the high occurrence of liver damage as an adverse drug reaction in the Chinese population,^27^ we included ‘risk of liver damage’ as an attribute.

Our research revealed that patients considered ‘risk of liver damage’ as the most essential attribute, whereas physicians ranked it the second most important after ‘reduction of LDL‐C level’. It is well documented that many drugs, including statins, anti‐inflammatory drugs and antitubercular drugs, can cause liver injuries.^28^ Considering the vital role of the liver in drug metabolism and overall human health, it is understandable why patients and physicians in our study placed significant emphasis on the risk of liver damage.

Overexposure to elevated blood LDL‐C levels poses a considerable risk for cardiovascular diseases, substantially reducing life expectancy.^29^ However, patients have shown much less concern regarding LDL‐C than physicians, possibly due to a lack of awareness of this as a major cardiovascular disease risk factor. A previous study reported a high prevalence of untreated and uncontrolled hyperlipidemia in China.^4^ Our findings suggested that insufficient attention to ‘reduction of LDL‐C level’ among patients may contribute to this issue. Therefore, continuous patient and public education are recommended to enhance awareness regarding the importance of ‘reduction of LDL‐C level’.

Numerous patient preference studies have consistently highlighted efficacy and safety as the most important attributes.^14^, ^30^ Our study confirmed and extended these findings, revealing that the mode and frequency of administration also influenced patient preferences, although to a lesser degree than the risk of liver damage. Similar results were observed in previous research studies on rheumatoid arthritis^17^ and osteoporosis^31^ patients, where oral and less frequent dosing regimens were favoured. Notably, we found the once‐every‐2‐weeks frequency was less favoured than the once‐a‐week frequency for patients. This may be attributed to biweekly dosing requiring recall of whether the drug was taken in the previous week, making it less convenient compared to a regular weekly dose.

Previous research has highlighted the high cost of PCSK9 inhibitors as a barrier to their widespread use.^32^ Our study revealed that while cost played a role in lipid‐lowering drug treatment decisions, it ranked fourth among physicians and fifth among patients among the six attributes considered. This may be due to the fact that the national drug price negotiation policy in China has included two PCSK9 inhibitors in the drug reimbursement list and reduced out‐of‐pocket costs. Our previous research showed that implementing price negotiation policy improved drug affordability,^33^ and our current study further supports this finding.

Moreover, we performed LCA to investigate preference heterogeneity, as evidenced by the significant estimated SD for the coefficients. The LCA revealed three distinct patient classes: focus on oral administration, focus on hepatic safety and frequency and focus on hepatic safety and cost. Patient age and self‐reported health status contributed to preference heterogeneity. Likewise, three physician classes were identified: frequency‐insensitive, efficacy‐focused and safety‐focused. This finding showed consistency with previous studies that have also identified patient preference heterogeneity in chronic low back pain treatment.^34^ This heterogeneity underscores the importance of individualizing treatment, considering the situation, preferences and other medical issues of patients.

Previous studies primarily concentrated on the comparative analysis of different lipid‐lowering drugs.^5^, ^35^, ^36^ Nevertheless, our DCE study is unique in that it evaluates the significance of these differences from the patient and physician perspectives. Despite the favourable efficacy, safety and administration frequency of PCSK9 inhibitors, our study found that subcutaneous injections were a source of distress for patients. Therefore, it can be cautiously inferred that orally administered lipid‐lowering drugs with good hepatic safety and efficacy will be preferred in the future. It is also noteworthy that further investigation is needed to assess the long‐term efficacy and safety of PCSK9 inhibitors, as their application is relatively recent. Statins, the classic lipid‐lowering drugs, remain indispensable.^37^

This research has several limitations. First, we recruit respondents from the patient organization and physician association, potentially influencing the representativeness of the sample. However, we compared the characteristics of the respondents with those of the patients nationwide, and we believe that the sample is still representative. Second, we only included six attributes which might overlook other factors that may potentially influence decision‐making. However, including too many attributes in the DCE might cause cognitive overload for respondents. Five or six attributes in DCE in health economics were the most common.^22^ Third, in our hypothetical scenario, we assumed no specific sequence in administrating lipid‐lowering drugs. This may not reflect actual clinical practice. We made this assumption to simplify the situation to focus on accurately determining preferences. Finally, the attributes and levels were established based on the current context in China. For instance, the minimum frequency of currently available lipid‐lowering drugs in China is monthly. Therefore, updated data on new lipid‐lowering drugs may change the study results.

## 5 CONCLUSIONS

The study revealed divergent preferences between patients and physicians regarding lipid‐lowering drug therapy. Patients prioritized hepatic safety, whereas physicians prioritized lowering LDL‐C levels. Patients valued oral administration and low frequency of administration more than physicians. Furthermore, we identified distinct preference patterns among physicians and patients, indicating the heterogeneity of preferences. This research highlighted the significance of shared decision‐making between Chinese physicians and patients regarding lipid‐lowering drug therapy due to the divergent preferences.

## AUTHOR CONTRIBUTIONS

**Lingli Zhang**: Conceptualization; investigation; data curation; methodology; validation; formal analysis; funding acquisition; writing—original draft. **Jiali Chen**: Methodology; data curation; investigation. **Zhaoliu Cao**: Investigation. **Mengdie Zhang**: Investigation. **Rui Ma**: Investigation. **Pei Zhang**: Investigation. **Guiqing Yao**: Writing—review and editing. **Xin Li**: Conceptualization; writing—review and editing; resources; funding acquisition; project administration; supervision; data curation.

## CONFLICT OF INTEREST STATEMENT

The authors declare no conflict of interest.

## ETHICS STATEMENT

This study was approved by the ethics committee of Nanjing Medical University, and all respondents provided written informed consent.

## Supporting information

Supporting information.

## Data Availability

The data are available from the corresponding author on reasonable request.
