# Supplementary material for: Patient versus physician preferences for lipid‐lowering drug therapy: A discrete choice experiment
Source: Health Expect. 2024 Apr 8;27(2):e14043. doi: 10.1111/hex.14043 (PMC11002318; doi:10.1111/hex.14043)
Supplement: Supplementary file 1 — Supporting information. [file HEX-27-e14043-s001.docx]

**SUPPORTING INFORMATION**

**Appendix 1** Literature search strategy

Search terms included (“dyslipidemia” or “hyperlipidemia” or “hypercholesterolemia” or “high blood cholesterol” or “antihyperlipidemic drug” or “lipid-lowering drug” or “PCSK9 inhibitor” or “statin”) in Title/Abstract. We searched PubMed and Web of Science for articles in English. The time window is from January 1, 2012, to April 30, 2022. Exclusion criteria for the literature were as follows: irrelevant with comparison and selection of different lipid-lowering drugs, no full-text publication available, and duplicate references.

**Appendix 2** Focus group discussions on candidate attributes

| **Candidate attributes** | **Several key opinions** | **Decision** |
| --- | --- | --- |
| Frequency of administration | Patient 1：I wish the medications were given less frequently, I can't remember to take them every day and miss them. | Include |
| Mode of administration | Patient 2：I don't like injections, they're not easy to carry.  Patient 3: It's better to take it orally than by injection. | Include |
| Reduction of LDL-C level | Physician 1：LDL-C is the most important lipid-lowering target. | Include |
| Reduction of triglyceride | Physician 2：TG levels are not as clinically important as LDL-C and more influenced by diet and other factors. | Exclude |
| Risk of myopathy | Physician 3：When using statins, we are asked to monitor the patient's creatinine kinase.  Patient 4: Muscle weakness and pain can affect my work. | Include |
| Risk of liver damage | Physician 4：Liver damage is the primary indicator of discontinuation.  Patient 5: Liver damage is so annoying. | Include |
| Risk of elevated blood glucose | Physician 5：The cause of elevated blood sugar is hard to pinpoint as a lipid-lowering drug. | Exclude |
| Risk of neurocognitive impairment | Physician 6：The main adverse reactions are liver damage and muscle complications, and other adverse reactions are of less concern to us and are rare. | Exclude |
| Skin problems at injection site | Patient 6: It's only the injectable drugs that have this problem, is this a duplicate of the dosing method above? | Exclude |
| Out-of-pocket monthly cost | Physician 7：We sometimes change the treatment plan depending on the patient's affordability.  Patient 7: I'd be a bit overwhelmed with medication costs over 500 Chinese yuan a month. | Include |

**Appendix 3** Reference of attribute and level

|  | Statins | Ezetimibe | Alirocumab | Evolocumab |
| --- | --- | --- | --- | --- |
| Frequency of administration^*^ | Once a day | Once a day | Once every two weeks | Once every two weeks or once a month |
| Mode of administration^*^ | Oral | Oral | Subcutaneous injection | Subcutaneous injection |
| Reduction of LDL-C level | 11% - 50% ^1^ | 18.58% ^2^ | 52.6% ^3^；56% ^4^; 55% ^5^ | 54.6% ^3^; 59% ^6^ |
| Risk of myopathy | 0.679% -1.674% ^7^; 1% - 5% ^8^ | 2% - 5% ^2^ | 0.5% ^5^; 2.0% ^3^ | 0.1% - 0.5% ^3^；0.7% ^6^ |
| Risk of liver damage | 0.602% - 2.133% ^7^; 0.5% - 3.0% ^9^; 3.67% or 5.83% ^10^ | 0 - 1% ^2^ | 2.3% ^5^; 0.9% ^3^ | 0.4% - 0.5% ^3^；1.8% ^6^ |
| Out-of-pocket ratio ^11^ | 0% (minimum) | 30% | 30% | 30% |
| Price per unit (CNY) | - | 1.5-6.8 (10mg) | 283.8 (140mg) | 306 (75mg) |
| Out-of-pocket monthly cost (CNY)^#^ | 0 (minimum) | 13.5-61.2 | 170.28 | 183.6 |

Abbreviation: CNY, Chinese yuan.

^*^ Frequency and mode of administration refer to drug insert, ^#^ A month was defined 30 days

**Reference**

1. Wadhera RK, Steen DL, Khan I, Giugliano RP, Foody JM. A review of low-density lipoprotein cholesterol, treatment strategies, and its impact on cardiovascular disease morbidity and mortality. *J Clin Lipidol*. 2016;10(3):472-489. doi:10.1016/j.jacl.2015.11.010

2. Pandor A, Ara RM, Tumur I, et al. Ezetimibe monotherapy for cholesterol lowering in 2,722 people: systematic review and meta-analysis of randomized controlled trials. *J Intern Med*. 2009;265(5):568-580. doi:10.1111/j.1365-2796.2008.02062.x

3. Zhang XL, Zhu QQ, Zhu L, et al. Safety and efficacy of anti-PCSK9 antibodies: a meta-analysis of 25 randomized, controlled trials. *BMC Med*. 2015;13:123. doi:10.1186/s12916-015-0358-8

4. Han Y, Chen J, Chopra VK, et al. ODYSSEY EAST: Alirocumab efficacy and safety vs ezetimibe in high cardiovascular risk patients with hypercholesterolemia and on maximally tolerated statin in China, India, and Thailand. *J Clin Lipidol*. 2020;14(1):98-108.e8. doi:10.1016/j.jacl.2019.10.015

5. Schwartz GG, Steg PG, Szarek M, et al. Alirocumab and cardiovascular outcomes after acute coronary syndrome. *N Engl J Med*. 2018;379(22):2097-2107. doi:10.1056/NEJMoa1801174

6. Sabatine MS, Giugliano RP, Keech AC, et al. Evolocumab and clinical outcomes in patients with cardiovascular disease. *N Engl J Med*. 2017;376(18):1713-1722. doi:10.1056/NEJMoa1615664

7. Tsui L, Ye P, Xu S, et al. Adverse drug reactions of statin therapy in China from 1989 to 2019: a national database analysis. *Eur J Hosp Pharm*. 2023;30(e1):e82-e89. doi:10.1136/ejhpharm-2022-003333

8. Stroes ES, Thompson PD, Corsini A, et al. Statin-associated muscle symptoms: impact on statin therapy-European atherosclerosis society consensus panel statement on assessment, aetiology and management. *Eur Heart J*. 2015;36(17):1012-1022. doi:10.1093/eurheartj/ehv043

9. McKenney JM, Davidson MH, Jacobson TA, Guyton JR, National lipid association statin safety assessment task force. final conclusions and recommendations of the national lipid association statin safety assessment task force. *Am J Cardiol*. 2006;97(8A):89C-94C. doi:10.1016/j.amjcard.2006.02.030

10. Sun Z, Yang W, Wang R, Liang L, Yi Z. Retrospective comparative study on the safety , economy and risk factors of the generic and branded atorvastatin calcium. *Chin J Hosp Pharm*. 2022;42(14):1447-1451. doi:10.13286/j.1001-5213.2022.14.10

11. National Healthcare Security Administration. Notice on the issuance of the national drug list for basic medical insurance, work injury insurance and maternity insurance (2021). Accessed July 6, 2022. http://www.nhsa.gov.cn/art/2021/12/3/art_37_7429.html

**Appendix 4** Prior parameters in the Bayesian D-optimal design

| Levels | Physician | | Patient | |
| --- | --- | --- | --- | --- |
|  | Prior mean | Prior variances | Prior mean | Prior variances |
| Frequency (every two weeks) | 0.185 | 0.042 | -0.093 | 0.115 |
| Frequency (once a day) | -0.194 | 0.030 | -0.540 | 0.070 |
| Frequency (once a month) | 0.181 | 0.035 | 0.513 | 0.099 |
| Mode(oral) | 0.228 | 0.006 | 0.625 | 0.021 |
| Reduction of LDL-C (low) | -0.723 | 0.021 | -0.506 | 0.060 |
| Reduction of LDL-C (high) | 0.627 | 0.016 | 0.570 | 0.057 |
| Risk of myopathy (0) | 0.424 | 0.022 | 0.093 | 0.056 |
| Risk of myopathy (2.5%) | -0.258 | 0.019 | -0.044 | 0.052 |
| Risk of liver damage (0) | 0.101 | 0.017 | 0.324 | 0.046 |
| Risk of liver damage (2.5%) | 0.242 | 0.016 | 0.027 | 0.045 |
| Out-of-pocket monthly cost (0 CNY) | 0.288 | 0.018 | 0.259 | 0.052 |
| Out-of-pocket monthly cost (100 CNY) | -0.014 | 0.017 | -0.214 | 0.059 |

Abbreviation: LDL-C, low-density lipoprotein cholesterol; CNY, Chinese Yuan.

**Appendix 5** Latent class analysis result of the patient

| Attribute and level | Class 1 | | Class 2 | | Class3 | |
| --- | --- | --- | --- | --- | --- | --- |
|  | Coefficient (95% CI) | P-value | Coefficient (95% CI) | P-value | Coefficient (95% CI) | P-value |
| Frequency of administration (ref: once a day) | | | | | | |
| Once a week | 1.00 (0.40, 1.60) | 0.001 | 0.62 (0.46, 0.77) | < 0.001 | -0.46 (-0.92, -0.01) | 0.05 |
| Once every two weeks | 0.80 (0.18, 1.41) | 0.01 | 0.53 (0.39, 0.67) | < 0.001 | -0.11 (-0.49, 0.28) | 0.59 |
| Once a month | 0.22 (-0.38, 0.83) | 0.47 | 0.88 (0.70, 1.06) | < 0.001 | 0.92 (0.39, 1.45) | 0.001 |
| Mode of administration (ref: oral) | | | | | | |
| Subcutaneous injection | -3.04 (-3.63, -2.44) | < 0.001 | -0.25 (-0.40, -0.11) | < 0.001 | -1.34 (-1.69, -0.98) | < 0.001 |
| Reduction of LDL-C level (ref: 10%) | | | | | | |
| 35% | -0.04 (-0.44, 0.36) | 0.85 | 0.67 (0.54, 0.79) | < 0.001 | -0.02 (-0.41, 0.37) | 0.92 |
| 60% | -1.00 (-1.48, -0.52) | < 0.001 | 0.67 (0.51, 0.83) | < 0.001 | 1.71 (1.25, 2.17) | < 0.001 |
| Risk of myopathy (ref: 0%) | | | | | | |
| 2.5% | -0.31 (-0.73, 0.11) | 0.15 | -0.06 (-0.17, 0.05) | 0.30 | -0.63(-0.94, -0.32) | < 0.001 |
| 5% | -0.25 (-0.62, 0.12) | 0.19 | -0.32 (-0.44, -0.21) | < 0.001 | -1.32(-1.70, -0.94) | < 0.001 |
| Risk of liver damage (ref: 0%) | | | | | | |
| 2.5% | 0.54 (0.03, 1.04) | 0.04 | -0.46 (-0.58, -0.34) | < 0.001 | -2.71 (-3.04, -2.38) | < 0.001 |
| 5% | 0.34 (-0.04, 0.71) | 0.08 | -0.93 (-1.09, -0.78) | < 0.001 | -3.31 (-3.79, -2.84) | < 0.001 |
| Out-of-pocket monthly cost | -0.0009 (-0.0036, 0.0017) | 0.48 | -0.0028 (-0.0035, -0.0021) | < 0.001 | -0.0093 (-0.0113, -0.0072) | < 0.001 |
| Patients within the class (N, %) | 114 (18.18) | | 410 (65.39) | | 103 (16.43) | |
| Model specification | Log likelihood | | -3949.264 | | | |
|  | AIC | | 7970.527 | | | |
|  | BIC | | 8244.811 | | | |

Abbreviation: ref, reference; LDL-C, low-density lipoprotein cholesterol; AIC, Akaike information criterion; BIC, Bayesian information criterion.

**Appendix 6** Latent class analysis result of the physician

| Attribute and level | Class 1 | | Class 2 | | Class3 | |
| --- | --- | --- | --- | --- | --- | --- |
|  | Coefficient (95% CI) | P-value | Coefficient (95% CI) | P-value | Coefficient (95% CI) | P-value |
| Frequency of administration (ref: once a day) | | | | | | |
| Once a week | -0.36 (-0.85, 0.13) | 0.15 | -1.07 (-1.71, -0.44) | 0.001 | 0.15 (-0.01, 0.31) | 0.06 |
| Once every two weeks | -0.16 (-0.71, 0.39) | 0.56 | -1.18 (-2.00, -0.36) | 0.005 | 0.15 (-0.01, 0.32) | 0.07 |
| Once a month | -0.23 (-0.84, 0.39) | 0.47 | 0.48 (-0.56, 1.52) | 0.36 | 0.21 (0.04, 0.38) | 0.01 |
| Mode of administration (ref: oral) | | | | | | |
| Subcutaneous injection | -0.61 (-0.95, -0.27) | < 0.001 | -1.23(-2.22, -0.24) | 0.015 | -0.11 (-0.22, 0.00) | 0.06 |
| Reduction of LDL-C level (ref: 10%) | | | | | | |
| 35% | 0.28 (-0.33, 0.89) | 0.36 | 3.23 (1.94, 4.51) | < 0.001 | -0.02 (-0.23, 0.19) | 0.87 |
| 60% | 1.14 (0.58, 1.70) | < 0.001 | 7.39 (5.39, 9.39) | < 0.001 | 0.04 (-0.14, 0.22) | 0.66 |
| Risk of myopathy (ref: 0%) | | | | | | |
| 2.5% | -1.52 (-2.05, -0.99) | < 0.001 | -2.61(-3.70, -1.52) | < 0.001 | -0.01 (-0.17, 0.16) | 0.94 |
| 5% | -1.66 (-2.09, -1.22) | < 0.001 | -1.74(-2.42, -1.07) | < 0.001 | -0.20 (-0.37, -0.03) | 0.02 |
| Risk of liver damage (ref: 0%) | | | | | | |
| 2.5% | -1.61 (-2.05, -1.16) | < 0.001 | -3.09(-4.28, -1.90) | < 0.001 | -0.32 (-0.45, -0.19) | < 0.001 |
| 5% | -2.71 (-3.26, -2.16) | < 0.001 | -2.91(-3.71, -2.12) | < 0.001 | -0.30 (-0.46, -0.14) | < 0.001 |
| Out-of-pocket monthly cost | -0.0068 (-0.0094, -0.0041) | < 0.001 | -0.0074(-0.0118, -0.0030) | 0.001 | -0.0008 (-0.0016, 0.0000) | 0.05 |
| Physicians within the class (N, %) | 52 (12.38) | | 102(24.29) | | 266 (63.33) | |
| Model specification | Log likelihood | | -2725.52 | | | |
|  | AIC | | 5523.04 | | | |
|  | BIC | | 5782.90 | | | |

Abbreviation: ref, reference; LDL-C, low-density lipoprotein cholesterol; AIC, Akaike information criterion; BIC, Bayesian information criterion.

**Appendix 7** Characteristics of three physician classes

| Characteristic | Class 1(n=52) | Class2(n=102) | Class3(n=266) | P-value |
| --- | --- | --- | --- | --- |
|  | No. (%) of physicians | No. (%) of physicians | No. (%) of physicians |  |
| Gender |  |  |  | 0.74 |
| Male | 24 (46.15) | 52 (50.98) | 124 (46.62) |  |
| Female | 28 (53.85) | 50 (49.02) | 142 (53.38) |  |
| Age |  |  |  | 0.70 |
| 18-30 | 16 (30.77) | 37 (36.27) | 91 (34.21) |  |
| 30-40 | 30 (57.69) | 60 (58.82) | 156 (58.65) |  |
| 40-50 | 6 (11.54) | 5 (4.90) | 17 (6.39) |  |
| 50-60 | 0 (0) | 0 (0) | 2 (0.75) |  |
| Education |  |  |  | 0.97 |
| Bachelor | 12 (23.08) | 30 (29.41) | 74 (27.82) |  |
| Master | 36 (69.23) | 63 (61.76) | 169 (63.53) |  |
| Doctor | 4 (7.69) | 9 (8.82) | 23 (8.65) |  |
| Academic title |  |  |  | 0.28 |
| Resident physician | 24 (46.15) | 32 (31.37) | 94 (35.34) |  |
| Attending physician | 18 (34.62) | 44 (43.14) | 116 (43.61) |  |
| Associate chief physician | 9 (17.31) | 19 (18.63) | 34 (12.78) |  |
| Chief physician | 1 (1.92) | 7 (6.86) | 22 (8.27) |  |
| Hospital level |  |  |  | 0.53 |
| Grade-A tertiary | 31 (59.62) | 46 (45.10) | 139 (52.26) |  |
| Grade-B tertiary | 9 (17.31) | 20 (19.61) | 47 (17.67) |  |
| Grade-C tertiary | 4 (7.69) | 13 (12.75) | 39 (14.66) |  |
| Secondary | 8 (15.38) | 20 (19.61) | 34 (12.78) |  |
| Primary | 0 (0) | 3 (2.94) | 7 (2.63) |  |
| Region |  |  |  | 0.24 |
| East | 31 (59.62) | 54 (52.94) | 161 (60.53) |  |
| Central | 9 (17.31) | 23 (22.55) | 64 (24.06) |  |
| West | 12 (23.08) | 25 (24.51) | 41 (15.41) |  |

**Appendix 8** Patients’ preference estimated with cost as a categorical variable

| Attribute and level | Coefficient (95%CI) | P-value | SD (95%CI) | SD P-value | Relative importance |
| --- | --- | --- | --- | --- | --- |
| Frequency of administration (ref: once a day) | | | | | 17.9% |
| Once a week | 0.96 (0.76, 1.17) | < 0.001 | 0.16 (-0.38, 0.70) | 0.57 |  |
| Once every two weeks | 0.81 (0.60, 1.02) | < 0.001 | 0.82 (0.43, 1.22) | < 0.001 |  |
| Once a month | 1.36 (1.10, 1.63) | < 0.001 | 1.26 (0.96, 1.55) | < 0.001 |  |
| Mode of administration (ref: oral) | | | | | 19.0% |
| Subcutaneous injection | -1.45 (-1.71, -1.18) | < 0.001 | 2.02 (1.77, 2.26) | < 0.001 |  |
| Reduction of LDL-C level (ref: 10%) | | | | | 14.1% |
| 35% | 0.85 (0.66, 1.03) | < 0.001 | 0.88 (0.61, 1.16) | < 0.001 |  |
| 60% | 1.08 (0.82, 1.33) | < 0.001 | 1.66 (1.37, 1.96) | < 0.001 |  |
| Risk of myopathy (ref: 0%) | | | | | 10.1% |
| 2.5% | -0.41 (-0.57, -0.25) | < 0.001 | -0.16 (-0.57, 0.24) | 0.43 |  |
| 5% | -0.77 (-0.95, -0.59) | < 0.001 | 0.84 (0.54, 1.15) | < 0.001 |  |
| Risk of liver damage (ref: 0%) | | | | | 23.9% |
| 2.5% | -1.16 (-1.37, -0.96) | < 0.001 | 1.64 (1.41, 1.87) | < 0.001 |  |
| 5% | -1.82 (-2.08, -1.56) | < 0.001 | 2.11 (1.79, 2.42) | < 0.001 |  |
| Out-of-pocket monthly cost (ref: 0 CNY) | | | | | 15.0% |
| 100 CNY | -0.42 (-0.60, -0.23) | < 0.001 | 0.98 (0.70, 1.27) | < 0.001 |  |
| 200 CNY | -1.14 (-1.40, -0.88) | < 0.001 | 2.03 (1.71, 2.35) | < 0.001 |  |
| Model specification | Log likelihood | | -4003.41 | | |
|  | AIC | | 8056.83 | | |
|  | BIC | | 8247.30 | | |

Abbreviation: ref, reference. LDL-C, low-density lipoprotein cholesterol; CNY, Chinese Yuan; AIC, Akaike information criterion; BIC, Bayesian information criterion.

**Appendix 9** Physicians’ preference estimated with cost as a categorical variable

| Attribute and level | Coefficient (95%CI) | P-value | SD (95%CI) | SD P-value | Relative importance |
| --- | --- | --- | --- | --- | --- |
| Frequency of administration (ref: once a day) | | | | | 6.2% |
| Once a week | 0.01 (-0.16, 0.18) | 0.90 | 0.11 (-0.27, 0.50) | 0.56 |  |
| Once every two weeks | 0.17 (-0.01, 0.36) | 0.06 | 0.27 (-0.15, 0.68) | 0.21 |  |
| Once a month | 0.23 (0.03, 0.43) | 0.02 | -0.67 (-0.98, -0.36) | < 0.001 |  |
| Mode of administration (ref: oral) | | | | | 7.8% |
| Subcutaneous injection | -0.29 (-0.43, -0.15) | < 0.001 | 0.75 (0.57, 0.93) |  |  |
| Reduction of LDL-C level (ref: 10%) | | | | | 33.5% |
| 35% | 0.43 (0.21, 0.65) | < 0.001 | 0.39 (-0.01, 0.80) | 0.06 |  |
| 60% | 1.24 (0.98, 1.49) | < 0.001 | 1.69 (1.42, 1.95) | < 0.001 |  |
| Risk of myopathy (ref: 0%) | | | | | 15.8% |
| 2.5% | -0.37 (-0.58, -0.16) | < 0.001 | 0.75 (0.52, 0.97) | < 0.001 |  |
| 5% | -0.58 (-0.78, -0.39) | < 0.001 | 0.85 (0.62, 1.07) | < 0.001 |  |
| Risk of liver damage (ref: 0%) | | | | | 25.8% |
| 2.5% | -0.68 (-0.85, -0.51) | < 0.001 | 0.28 (-0.17, 0.73) | 0.23 |  |
| 5% | -0.95 (-1.16, -0.75) | < 0.001 | 0.84 (0.60, 1.09) | < 0.001 |  |
| Out-of-pocket monthly cost (ref: 0 CNY) | | | | | 10.9% |
| 100 CNY | -0.09 (-0.24, 0.06) | 0.23 | -0.43 (-0.71, -0.16) | 0.002 |  |
| 200 CNY | -0.40 (-0.60, -0.21) | < 0.001 | 0.71 (0.45, 0.97) | < 0.001 |  |
| Model specification | Log likelihood | | -2845.53 | | |
|  | AIC | | 5741.06 | | |
|  | BIC | | 5921.52 | | |

Abbreviation: ref, reference; LDL-C, low-density lipoprotein cholesterol; CNY, Chinese Yuan; AIC, Akaike information criterion; BIC, Bayesian information criterion.

**Appendix 10** Relative importance of attributes for patients grouped by age

Abbreviation: LDL-C, low-density lipoprotein cholesterol.
